# ‘We all need to be on the same page’: sustainment of healthy food retail practices in Australian public settings

**DOI:** 10.1093/heapro/daag092

**Published:** 2026-07-14

**Authors:** Bettina Backman, Shaan S Naughton, Serene Yoong, Victoria Brown, Miranda R Blake

**Affiliations:** Deakin Institute for Health Transformation, Deakin Centre for Global Preventive Health and Nutrition, School of Health and Social Development, Faculty of Health, Deakin University, 221 Burwood Highway, Burwood, VIC 3125, Australia; Deakin Institute for Health Transformation, Deakin Centre for Global Preventive Health and Nutrition, School of Health and Social Development, Faculty of Health, Deakin University, 221 Burwood Highway, Burwood, VIC 3125, Australia; Deakin Institute for Health Transformation, Deakin Centre for Global Preventive Health and Nutrition, School of Health and Social Development, Faculty of Health, Deakin University, 221 Burwood Highway, Burwood, VIC 3125, Australia; Deakin Institute for Health Transformation, Deakin Centre for Global Preventive Health and Nutrition, School of Health and Social Development, Faculty of Health, Deakin University, 221 Burwood Highway, Burwood, VIC 3125, Australia; Deakin Health Economics (DHE), Deakin Institute for Health Transformation, Deakin University, 221 Burwood Highway, Burwood, VIC 3125, Australia; Deakin Institute for Health Transformation, Deakin Centre for Global Preventive Health and Nutrition, School of Health and Social Development, Faculty of Health, Deakin University, 221 Burwood Highway, Burwood, VIC 3125, Australia

**Keywords:** food services, food environment, public policy, health promotion, implementation science, program sustainability

## Abstract

Sustained policy-driven changes to the availability and promotion of healthier food and drinks in foodservice outlets are essential to support population health over time. Limited research has explored whether why and how healthy food retail practices become easier or more challenging to maintain over time. This study aimed to explore food retailer (managers, staff, and volunteers) and implementation support practitioner (e.g. dietitians and health promotion practitioners) experiences with and perspectives on the (i) feasibility of, (ii) key determinants of, and (iii) strategies for sustaining healthy food retail practices in a range of Australian public settings (e.g. healthcare facilities). Semi-structured interviews were conducted with 17 participants (nine practitioners and eight retailers) representing food outlets with experience sustaining healthy food retail practices in sport and recreation centres, schools, and healthcare facilities across six Australian jurisdictions. Interviews were thematically analyzed, using primarily a deductive approach informed by the Integrated Sustainability Framework, sustainment-explicit Expert Recommendations for Implementing Change glossary, and marketing mix theory. Key determinants of sustainment (Theme 1) were related to ‘intervention-context fit and outlet capacity’; ‘people and processes driving sustainment’; and ‘top-down leadership and systemic support’. Multiple interconnected strategies to support sustainment (Theme 2) were identified, including ‘iterative, evaluative, and adaptive strategies to support fit and fidelity’; ‘engagement and behaviour-focused strategies to build capacity and demand’; and ‘embedding healthy food retail within organizational governance and policy systems’. Sustaining healthy food retail practices in public settings is a dynamic process, requiring consistent alignment between inner and outer context, support systems, and engagement with key actors.

Contribution to Health Promotion

- One of the first studies to explicitly explore how healthy food retail practices are sustained over time.
- Sustaining healthy food retail practices depends on factors at multiple socioecological levels (e.g. individual, organizational, and policy), involving different actors (e.g. retailers, health promotion practitioners, governments, suppliers).
- Ongoing monitoring, adaptation, upskilling, and clear leadership can help support long-term sustainment.
- Findings highlight the importance of continued organizational and policy support to maintain healthier food environments.

## Background

Sustained changes to physical and social environments are essential to support population health over time, including changes to food environments in public settings. Foodservice outlets, where foods and drinks are purchased for immediate consumption (Hillier-Brown *et al*. 2017), play a growing role in influencing dietary behaviours due to increasing reliance on out-of-home food and drink consumption (Adams *et al*. 2015, Calba 2024). Foodservice outlets can be found in public (i.e. government-owned) settings such as schools (Lawlis *et al*. 2016, Coady *et al*. 2025); healthcare facilities (James *et al*. 2017, Tsai *et al*. 2018); and sport and recreation centres (Westberg *et al*. 2022, Riesenberg *et al*. 2023) in many high-income countries. Governments in these countries often regulate food environments in public settings through policies and guidelines aimed at improving the healthiness (i.e. nutritional quality) of foods and drinks available and promoted, to support overall public health (L'Abbé *et al*. 2013, Backman *et al*. 2026a). However, evidence from different contexts, including community sporting settings in Australia (Boelsen-Robinson *et al*. 2017) and Canada (Olstad *et al*. 2015); healthcare facilities in Australia (Tsai *et al*. 2018); Canada (Kennedy *et al*. 2025); New Zealand (Mackay *et al*. 2024); and the UK (James *et al*. 2017) and schools in Australia (Reilly *et al*. 2021) and New Zealand (Pillay *et al*. 2025), suggests that existence of a policy alone does not guarantee its implementation in practice. To address this policy-implementation gap, implementation support practitioners (hereafter ‘practitioners’) such as dietitians and health promotion officers often support healthy food retail policy implementation, acting as intermediaries between policy and practice and providing technical assistance and supervision through governing organizations (e.g. healthcare settings, local governments) or external health promotion organizations (Rosewarne *et al*. 2022).

Government healthy food retail policies are commonly underpinned by the ‘4 Ps of marketing’ (product, placement, price, and promotion) framework (NHS England 2022, Backman *et al*. 2025) reflecting strong evidence that these practices can influence consumer purchasing decisions at the food outlet level (Cameron *et al*. 2016, Hillier-Brown *et al*. 2017, Mah *et al*. 2019). Such policies typically strongly recommend or mandate changes to one or more of the in-store marketing practices to support healthier customer food and drink purchases (hereafter referred to as ‘healthy food retail practices’) (Backman *et al*. 2026a), with compliance requirements focused on meeting prescribed targets for higher proportions of healthier relative to less healthy products (Rosewarne *et al*. 2020). The long-term influence of healthy food retail practices on purchasing behaviour and associated health outcomes depends on these practices being *sustained* over time (Slapø *et al*. 2022).

Sustainment, in this study, refers to the continued delivery of healthy food retail practices following their initial implementation (Moore *et al*. 2017). Evidence suggests that sustainment of health interventions more generally is influenced by multiple determinants across socio-ecological levels, from outer context (e.g. sociopolitical environment) to organizational and individual (e.g. customers, retailer and staff) characteristics, behaviour, and processes (Shelton *et al*. 2018). Targeted sustainment strategies (e.g. staff training) used by governments, organizations (e.g. public institutions) or individuals (e.g. practitioners) are often required to address these determinants (Nathan *et al*. 2022). The determinants and strategies of sustained settings-based health promotion interventions have received increased attention in recent years (Hall *et al*. 2023), particularly in school and childcare contexts (Shoesmith *et al*. 2021). These reviews underscore the importance of inner contextual factors for sustainment, including leadership support and organizational resources (Shoesmith *et al*. 2021).

However, evidence on the long-term (>2 years) sustainment of healthy food retail practices in foodservice settings remains limited (Cameron *et al*. 2016, Gupta *et al*. 2022, Backman *et al*. 2026b). While longer-term intervention follow-ups are increasingly reported in grocery and convenience store contexts, particularly in North America (Backman *et al*. 2026b), foodservice research is sparse and largely limited to short-to-medium-term evaluations (<2 years) in specific settings such as sport and recreation centres (Olstad *et al*. 2019, Blake *et al*. 2021). Shorter follow-ups constrain understanding of how sustainment is achieved over time, primarily capturing implementation or predicted sustainment challenges rather than actual longer-term sustainment mechanisms. A small number of studies report longer-term healthy food retail intervention follow-ups in settings including sport and recreation (Blake *et al*. 2022); hospitals (Law *et al*. 2021, Rosin *et al*. 2024); and schools (Pettigrew *et al*. 2018, Levay *et al*. 2019) However, these studies rarely explicitly distinguish determinants of initial implementation (e.g. anticipated financial implications) from those influencing longer-term sustainment (e.g. actual financial implications) (Levay *et al*. 2019, Law *et al*. 2021, Rosin *et al*. 2024). Where sustainment outcomes are reported, the determinants of sustainment are often treated as secondary findings (Pettigrew *et al*. 2018) or not reported (Blake *et al*. 2022).

To our knowledge, determinants of sustainment in foodservice context have not been previously examined using established implementation science frameworks, and little is known about which sustainment strategies are perceived as effective and relevant in practice. Identifying common determinants across contexts enables the design of scalable sustainment support structures. Although healthy food retail policies in Australia differ across states and settings, they share common foundations in national nutrition policies (Backman *et al*. 2026a) such as the Australian Dietary Guidelines (National Health and Medical Research Council 2013). This study therefore aimed to explore retailer (as responsible for day-to-day operations and ongoing policy compliance) and practitioner (as those supporting implementation and overseeing compliance) experiences with and perspectives on the sustainment of healthy food retail practices in Australian public foodservice settings. Specifically, it explored (i) feasibility of sustaining healthy food retail practices over time; (ii) key determinants influencing sustainment; and (iii) the strategies and support needs considered important for maintaining healthy food retail practices in public settings.

## Methods

Semi-structured interviews and applied thematic analysis were used to explore retailer and practitioner experiences of sustaining healthy food retail practices in Australian public foodservice settings. A pragmatic applied qualitative approach was adopted to generate policy- and practice-relevant evidence (Guest *et al*. 2012). This study is reported in accordance with the Consolidated Criteria for Reporting Qualitative Research checklist (Tong *et al*. 2007).

### Participants and recruitment

Participants were purposively recruited from public settings where state government healthy food retail policies commonly influence food and drink offerings, including schools, healthcare, and community (non-elite) sport and recreation settings (Backman *et al*. 2026a). Sampling aimed to include participants from at least three Australian states and territories. Recruitment also sought balance between metropolitan and regional locations, and retailer and practitioner perspectives. Priority was given to food outlets that had implemented healthy food retail practices at least 2 years prior to interview, although participants with shorter sustainment experience were also eligible if they could reflect on maintaining changes over time.

Eligible participants were aged 18 years or older and included retailers, managers, food outlet staff, or volunteers with responsibility for implementing and/or maintaining healthy food retail practices and practitioners (e.g. dietitians, nutritionists, and health promotion practitioners) involved in supporting implementation and sustainment. Recruitment occurred April–August 2025 through (i) professional networks and partner organizations, (ii) direct contact with food outlets or governing organizations using publicly available details, and (iii) dissemination of study invitations via newsletters, LinkedIn, direct email, and related research activities. Snowball sampling was also used. Interested participants registered via a brief Qualtrics survey (Supplementary File S1), after which the lead author confirmed eligibility and arranged interviews. Recruitment continued until thematic sufficiency was reached (Hennink and Kaiser 2022), whereby additional interviews yielded no substantively new insights to the study aims. Approximately half of the contacted participants or organizations responded to the recruitment invitations. No participants who agreed to participate withdrew from the study.

### Data collection

Single, semi-structured, in-depth interviews were conducted via online videoconferencing (Zoom or Microsoft Teams) by the lead author (female, PhD candidate at the time of the study, experienced in qualitative interviewing). Interviews averaged 47 minutes (range 35–60 minutes) and were primarily conducted individually, with one joint interview involving two retailers who shared a management role within the same setting. Participants received a AUD$100 electronic gift voucher in recognition of their time. An interview guide (Supplementary File S2) with open-ended questions explored: (i) participant and food outlet context; (ii) experiences of sustaining healthy food retail practices over time, including perceived feasibility, barriers, and enablers; and (iii) strategies used or perceived as necessary to support long-term sustainment. The guide was not pilot tested but was iteratively refined during data collection. All interviews were audio-recorded and transcribed using platform-based auto-transcription. Transcripts were checked for accuracy by the lead author. Participants were offered the opportunity to review their transcript for accuracy and clarification prior to analysis. Eleven participants completed this, with five requesting transcript edits (mostly minor wording refinements and one more substantive clarification).

### Data analysis

Interview data were analyzed in NVivo version 14 (Lumivero 2023) using applied thematic analysis, as described by Guest *et al*. (2012). A hybrid deductive-inductive coding approach was used (Fereday and Muir-Cochrane 2006), guided by a multi-theory framework integrating concepts from implementation science and food retail (Supplementary File S3). This framework combined an Integrated Sustainability Framework (ISF) (Shelton *et al*. 2018), adapted to food retail by Backman *et al*. (2026b), to identify healthy food retail specific sustainment determinants, the sustainment-explicit Expert Recommendations for Implementing Change (ERIC) taxonomy to classify sustainment strategies (Nathan *et al*. 2022), and the Marketing Mix (Goi 2009) to categorize healthy food retail practices. The ISF and ERIC domains and codes were iteratively re-arranged and renamed during the analysis to better reflect the food retail context.

Sustainment related sections of the first interview were coded independently by three authors (BB, MRB, and SN), and three additional transcripts were double-coded. After refining code definitions, the remaining transcripts and contextual information of all transcripts were coded by the lead author, with ongoing refinement of the codebook as analysis progressed. Coding was primarily semantic, capturing participants’ explicit experiences and perspectives, with limited latent interpretation applied where relevant. Initial themes were developed by analyzing how coded data aligned with the study aims and were iteratively refined by the lead author and reviewed by all authors. Final themes and sub-themes were supported with illustrative participant quotations.

### Reflexivity

The research team combined backgrounds in public health, nutrition, and implementation science with prior experience in healthy food retail research. These perspectives informed data interpretation. Reflexivity was maintained through ongoing critical reflection and regular team discussions during study conception, data collection, and analysis to identify and address potential biases.

### Ethics

Lower-risk ethics approval was granted by Deakin University Human Research Ethics Advisory Group (2025/HE000042 and HEAG-H 92_2023). All participants provided informed consent via an online Qualtrics survey (Supplementary File S1), with consent reconfirmed verbally at the start of each interview.

## Results

Seventeen participants (nine practitioners and eight retailers) were interviewed between May–July 2025 (Table 1). Participants were primarily from Victoria (53%) and most represented sport and recreation settings (59%). Most states and territories (excluding Australian Capital Territory and Tasmania) were represented, with a mix of metropolitan and regional settings. Participants represented or worked in food outlets across 10 governing organizations, including local governments (*n* = 3), state government health promotion agencies (*n* = 3), public schools (*n* = 2), a non-governmental health promotion organization (*n* = 1), and a local health district (*n* = 1). In three cases, both a practitioner and retailer within the same governing organization participated in separate interviews. One participant represented a national food business not tied to a single governing organization. Most participants (*n* = 14) represented food outlets that were operating under state-level healthy food retail policies, including four in settings where these policies were mandatory (schools and/or healthcare, depending on the jurisdiction). Others (*n* = 3) represented outlets that had implemented practices based on healthy food retail policies for other settings or jurisdictions.

**Table 1:** Participant characteristics from qualitative interviews exploring the long-term sustainment of healthy food retail practices in Australian public foodservice settings (*n* = 17).

Participant characteristics and food outlets represented	*n*	%
**Participant roles**		
***Retailers***	**8**	**47%**
Food business or outlet manager or similar role	6	35%
Food outlet volunteer	1	6%
Marketing role	1	6%
***Practitioners***	**9**	**53%**
Health promotion practitioner	6	35%
Dietitian/nutritionist	2	12%
Other	1	6%
**Characteristics of the food outlets represented**		
***Jurisdiction***		
Victoria	9	53%
Queensland	2	12%
Western Australia	2	12%
New South Wales	1	6%
Northern Territory	1	6%
South Australia	1	6%
National (multiple states)	1	6%
***Regional vs metropolitan***		
Metropolitan	7	41%
Regional	6	35%
Both	3	18%
Unclear	1	6%
***Settings represented*** *^a^*		
Sport and recreation	10	59%
School	6	35%
Healthcare	3	18%
Private^b^	2	12%
***Food outlet types represented*** *^a^*		
Café/cafeteria	8	47%
Canteen	9	53%
Kiosk	8	47%
Vending machines	3	18%
Gift shop	2	12%
Food trucks	1	6%
***Staff types within food outlet (paid vs volunteer)***		
Paid staff (only)	9	53%
Mix of paid staff and volunteers	7	41%
Volunteers (only)	1	6%

^a^Practitioners and some retailers worked across multiple settings and food outlet types.

^b^Private settings refer to food outlets located outside government-owned public settings. While outside the scope of this study, these settings are represented because some participants worked across both public and private settings.

Café/cafeteria = counter-service outlets selling drinks, meals, and snacks (e.g. coffee and sandwiches) for immediate consumption or takeaway; Canteen = food outlets selling meals, snacks, and drinks for a defined population within a specific setting (e.g. school and sporting centre); Kiosk = small outlets with a limited product range, typically focused on snacks, drinks, or light meals; Vending machines = automated/self-service machines selling packaged snacks, drinks, or light meals; Gift shops = retail outlets primarily selling non-food items, with a limited food or drink offering; Food trucks = mobile food outlets preparing and selling ready-to-eat foods from a vehicle.

### Healthy food retail experience

Most participants (*n* = 12; 71%) represented organizations with more than 2 years of experience sustaining healthy food retail practices, several with six or more years of experience (up to 17 years). The remaining five participants represented organizations with between 1 and 2 years of sustainment experience. All participants reported increasing the availability of healthier foods and drinks. Most also described reducing less healthy options (*n* = 14), changing product placement (*n* = 13), and using promotional strategies (*n* = 12), while pricing strategies were less common (*n* = 7). School-based implementers additionally described practices such as setting serving size limits and restricting times when less healthy items could be purchased.

### Thematic findings

**Figure 1:**
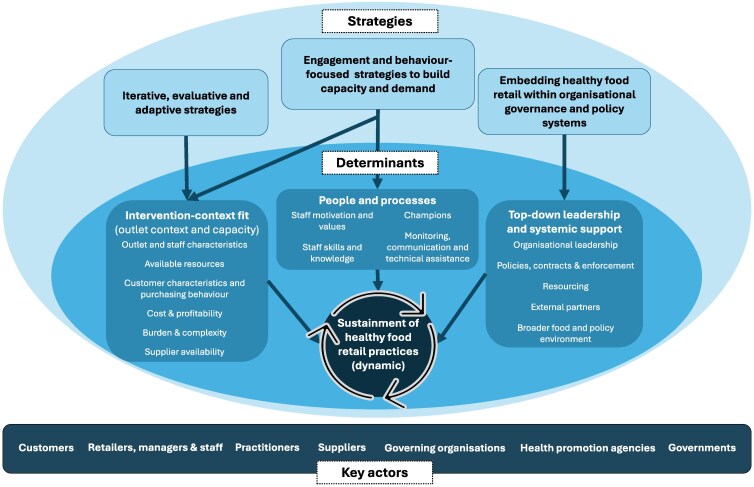
Thematic framework derived from the interview findings, illustrating key determinants, supporting strategies and the different actors involved in the sustainment of healthy food retail practices in Australian public settings.

The thematic findings focus primarily on sustainment of changes to product availability, reflecting the practices most discussed by participants. Two interrelated main themes were developed to describe how sustainment was perceived and understood (determinants) and supported (strategies). These findings are summarized and illustrated in Fig. 1.

#### Theme 1: What makes sustainment easy and what makes it challenging

Participants described sustainment as an ongoing, dynamic process which was described as both easy and difficult, often simultaneously depending on the healthy food retail practices being implemented. For example, maintaining a higher proportional availability of healthier drinks relative to less healthy drinks was considered easier compared to sustaining changes to food availability. Practitioners often described sustainment of any healthy food retail practice as generally challenging and highly context (setting and outlet staff) dependent, with some outlets more successful than others. Retailers generally expressed greater optimism, reporting that sustainment of healthy food retail practices was ‘not so difficult’ once changes were successfully implemented. One retailer described the challenges of implementing healthier product offerings, noting that sustaining these changes was easier:

Making the changes is hard, because you always see a drop in revenue. When we first removed the sugary soft drinks, we saw a huge drop in revenue, but over time, it’s built up again… Maintaining it is not difficult. We’ve got systems in place

(Retailer 1, national, multiple settings)

There were some differences in how sustainment was conceptualized or operationalized between retailers and practitioners. Retailers operating under policies enforced by the state government or their governing organization focused on sustainment of compliance-related practices, such as percentage targets for healthier items. Practitioners, in contrast, often emphasized sustainment of broader organizational capacity and commitment, retailers’ capability and willingness to continue healthy practices, and practitioners’ own capacity to provide ongoing oversight and support. They also tended to view sustainment through a longer-term lens, focusing on whether healthy food retail practices remained despite changing priorities or reduced support. Many retailers did not initially frame their experience as ‘long-term sustainment’ as they had accepted the practices as the new normal. These retailers often focused on shorter-term implementation and fidelity-related challenges, such as maintaining the required daily proportional availability of healthier offerings, reflecting how implementation and sustainment often overlap in practice.

There's [healthy] food on offer, but it sells so quickly, but I just don't have the staff…. I don't think I'll be able to get budget for more staffing [to prepare healthier options]. That's where it comes into the suppliers and the frozen sandwiches. At those times when we've made all the fresh food and that's all sold out, how do we restock? That's why we need to resort to having something that was frozen, ready to go. So, that's probably been a little bit of a challenge, and it still is, but we're working through it.

(Retailer 2, sport and recreation, Victoria, regional)

In reflecting on what makes sustainment easy or challenging, participants described multiple, interacting determinants influencing the sustainment of healthy food retail practices across all domains of the adapted ISF. These determinants are organized into three sub-themes, described below.

##### Sub-theme 1.1: Intervention-context fit: alignment with food outlet context and capacity

Sustainment was often described as dependent on the fit between healthy food retail practices and the outlet or organizational context. ‘Fit’ reflected alignment with organizational priorities, operational capacity, business models, and customer expectations. For example, outlets relying on external lease holders, volunteers, or limited staffing were seen as more vulnerable to discontinuing healthy practices, particularly where there was insufficient practitioner capacity to monitor compliance or support food outlets (see Sub-theme 1.2). Sustainment was perceived to be easier when healthy food provision aligned with organizational culture, mission, and internal policies, creating shared expectations and normalizing healthier practices over time.

I hope that it [healthy food provision] continues down this track… I mean I'm only guessing that it's moving into an area where this is the expectation of what we stock.

(Retailer 3, sport and recreation, Victoria, regional)

Financial and customer considerations were closely linked to intervention-context fit. Rising ingredient costs and higher profit margins on less healthy products challenged ongoing viability, although retailers noted that healthier items often performed well once customer-acceptable options were identified. Ongoing review of sales data and customer feedback was perceived as beneficial for determining whether practices were viable in the long term, highlighting the essential role of customer purchasing behaviour in sustainment. Outlets preparing house-made items also described higher ongoing workloads and costs associated with healthier options, whereas pre-packaged products were considered lower-burden and a good fit for most contexts. However, difficulties sourcing suitable healthier alternatives from suppliers made it challenging to introduce new healthier items and maintain product availability targets when menus were periodically updated.

At this point in time, maintaining it [policy targets] with what we have now, will not be a problem. We have already found the suppliers that we need. But if we were to introduce more [healthier] items, it'll be tough if we found anything new [to implement].

(Retailer 4, sport and recreation, Victoria, metropolitan)

##### Sub-theme 1.2: People and processes driving sustainment

Food outlet staff and manager motivation, values, and attitudes were seen as critical to making healthy food retail the ‘new normal’ given their influence on daily outlet operations. Participants noted that sustainment was easier when outlet managers and staff viewed healthy food retail as aligned with their values, whereas managers prioritizing commercial outcomes or with less healthy food preferences themselves were often more resistant to reducing less healthy options. This contrast was often brought up by practitioners working with a range of food outlets:

The challenges really are if there's no policy in place within the organisation [to support sustainment]. That is a challenge for any new staff coming on board. It can be a challenge, but we've also seen that it [new staff] can also be a win depending on who the canteen manager or the person in that leading role to run the canteen, depending on what their values are.

(Practitioner 1, sport and recreation, Western Australia, metropolitan)

Manager and staff knowledge, skills, and confidence in implementing healthy food practices were further considered to influence sustainment. Practitioners often questioned retailers’ capacity and capability to accurately classify products, while retailers generally felt confident or indicated that they had access to supporting resources (e.g. classification tools) if needed. Some practitioners and retailers described tensions when retailer perceptions of ‘healthier’ items did not align with formal food and drink classification systems, contributing to frustration and resistance. As one practitioner described:

It's not only understanding the guidelines. It's actually a lot of the time, fundamentally understanding the rationale and disagreeing with the rationale. I've had a hospital auxiliary women scream at me and accuse me of causing cancer in them, because they're forced to drink Diet Coke, because it's not sugar-sweetened, whereas there's nothing wrong with normal Coke.

(Practitioner 2, healthcare and schools, New South Wales, regional)

Practitioners and other ‘healthy food retail champions’ (e.g. dedicated food outlet staff member) were seen as key players coordinating sustainment and associated processes and liaising with other key actors (e.g. different organizational departments, suppliers). Key processes that participants described as essential for sustainment included clear communication (between key actors), documentation, monitoring, and technical assistance. Monitoring and reporting requirements, particularly where mandated by governing organizations or state policies, reinforced accountability and retailer motivation, although retailers without such requirements viewed sustainment as more dependent on personal or food outlet’s commitment.

…The [school] canteen is obliged to offer healthy options each day, but those requirements and obligations aren't on sporting associations… it's something that we've opted to do, not that we're obliged to do.

(Retailer 5, sport and recreation, Queensland, metropolitan)

##### Sub-theme 1.3: Top-down leadership and systemic support

Leadership from governing organizations, reinforced through formal policies and accountability mechanisms (e.g. monitoring and contractual requirements), was viewed as a key enabler. Similarly, regulatory and policy requirements at state or local government level were perceived to strengthen sustainment by creating consistent expectations across food outlets and reducing reliance on outlet-level motivation. However, participants noted that weak or inconsistent leadership and enforcement can increase reliance on practitioners and outlet managers, risking healthy food retail practices being deprioritized or discontinued in the long term. A national retailer also highlighted challenges associated with navigating inconsistent state-based policies, particularly for organizations operating across multiple jurisdictions:

When we talk to our suppliers, the very first question when we try to negotiate pricing, they say, ‘Well, how much of this are you going to buy?’ And if the policy was nationwide, we can say, ‘Well, we'll buy 10,000 of that product’. But because it's only compliant [considered as healthier item] in some states and not others, it reduces our purchasing power [affecting long term financial viability].

…And we've seen the changes in the Victorian policy that's become stricter over time. Same as WA, Queensland as well. So yeah, we've seen a lot of changes in those policies which has made it challenging.

(Retailer 1, national, multiple settings)

External partners, including health promotion organizations, were viewed as important supporters, providing coordination, tools and resources (e.g. food classification databases), and access to funding, strengthening broader system-level sustainment support. Participants emphasized the need for suppliers and food manufacturers to be aligned with healthy food retail goals to support access to affordable, context-appropriate packaged items. The higher cost of healthier items was also identified as a challenge to ongoing viability:

The cost, because unfortunately, fruit and vegetables, they are more expensive… we're happy to cover our costs when we're not-for-profit. But we certainly don't want to be losing money, because then we wouldn't be open five days a week for long.

(Retailers 6, school, Victoria, metropolitan)

While some retailers reported that small price increases were accepted by customers, others raised concerns that higher prices could disproportionately affect families from lower socioeconomic backgrounds:

The lower socio-economic families… Especially when you’ve got multiple kids. Imagine if you just changing to a healthier choice and everything [food prices] have doubled. It’s a lot when you’re doing it by child.

(Retailer 8, school, Western Australia, unclear regionality)

#### Theme 2: Strategies supporting sustainment

Participants described a range of interconnected strategies that supported the long-term sustainment of healthy food retail practices, across all ERIC domains. Key healthy food retail specific sustainment strategies are described in Supplementary File S4, with thematic findings summarized below.

##### Sub-theme 2.1: Using iterative, evaluative, and adaptive strategies to support intervention fit and fidelity

Participants acknowledged that sustainment requires ongoing evaluation, feedback, and adaptation to ensure alignment with changing operating conditions. Monitoring and evaluation were among the most frequently reported sustainment strategies, serving both as accountability and improvement mechanisms enabling adaptation over time. Product availability audits were commonly used to supervise outlets and provide feedback, and in some policy contexts, audits also functioned as formal compliance reporting to governing bodies. Practitioners emphasized that routine monitoring was necessary to prevent discontinuation, while retailers noted that audits created an incentive to sustain practices. Participants highlighted the importance of quality monitoring systems, including easy-to-use audit tools and pre-determined auditing frequency. While more frequent monitoring was perceived as more effective, capacity constraints often limited this. Views differed on whether monitoring should be retailer- or practitioner-led. Most practitioners were concerned about the reliability of self-reporting, while some retailers preferred self-audits to avoid external errors affecting compliance ratings.

Sustainment was also supported through adaptation processes. For example, tailoring practices to local capacity and resources was seen as critical for reducing burden and improving sustainment feasibility. Staff and consumer feedback were perceived essential for informing ongoing menu updates, while staged implementation, such as small trials and taste testing, helped identify products that customers might continue to purchase over time:

Retailer 6: ‘Over the years, we have tried other [healthier] items. Vegetable burgers, that didn't go so well, vegetable patties… It's a matter of trial and error, and having these taste testing days and working out what's good, and sticking with items that are good. Because once they [customers/students] know what it is, they [healthier options] really start to take off.’

Retailer 7: ‘And the kids actually get a say. They have taster sessions where the kids can try it. If they really enjoy it, that's how we can put it on the menu.’

(Retailers 6 and 7, school, Victoria, metropolitan)

##### Sub-theme 2.2: Building healthy food retail capacity and demand through engagement and behaviour-focused strategies

Retailers emphasized the importance of continuously increasing customer demand for healthier options through ongoing promotion and convenience (e.g. ensuring healthier options are ‘grab-and-go’) so that healthier items stay financially viable. Practitioners also emphasized other people-focused strategies, including the importance of building retailer and staff capacity through training. Both retailers and practitioners highlighted the value of embedding training into staff induction, helping to prevent knowledge loss due to staff turnover. Implementation support tools, such as product layout planograms and food healthiness classification tools, were described as helping reduce reliance on frequent practitioner or external support. However, practitioners noted that uptake of these tools into routine practice often took time and varied across outlets, depending on factors such as manager and staff characteristics and capacity. As one practitioner explained:

That's one of the requirements of the contract that they [retailers] submit their assessed menu. So, they have to have done the FoodChecker report and send that to us and then I just check that over just to make sure that's correct… The facility that is meeting the guidelines is doing really well and doing that themselves.

…We have two different categories for the facilities. Category A facilities, the facilities with paid food service staff, category B are the facilities with just volunteer staff… I heavily support the category B facilities. I go in and I'll conduct the FoodChecker report with them. Trying to teach them.

(Practitioner 4, sport and recreation, Victoria, regional)

Ongoing supervision and tailored technical assistance were also described as important, particularly for volunteer-run outlets or those with weaker leadership support. Dedicated support roles, such as health promotion practitioners or dietitians within councils or governing organizations, were described as central points of contact, providing supervision and practical support (e.g. audits, product classification, supplier sourcing, and policy interpretation). Practitioner roles were frequently described as someone driving implementation and sustainment efforts (i.e. ‘sustainment champions’), although in some contexts this role was also held by or shared with motivated outlet managers or staff. Champion roles were considered most effective when they included dedicated time and organizational support (see sub-theme 2.3).

Retailers or the health services are meant to be doing this work themselves. They're meant to be classifying the recipes, classifying their products. But oftentimes, because in Queensland, we have no funded capacity at a local level to do this work, they will come to me and say, ‘Can you do this for me?’ …If a health service has a dietitian, for example, that has good sort of nutrition and food knowledge that has been allocated this workload, then they will do it more independently.

(Practitioner 5, healthcare, Queensland, metropolitan and regional)

##### Sub-theme 2.3: Embedding healthy food retail within organizational governance and policy systems

Practitioners and retailers emphasized the importance of embedding healthy food retail priorities across multiple governance levels to create a more consistent operating environment and reduce reliance on retailer motivation or time-limited projects. Leadership endorsement at the governing organization level was described as a strategy to keep healthy food retail on organizational agendas. Formalizing healthy food retail practices through policies and contracts by governing organizations was viewed as a cornerstone sustainment strategy to shift the focus from an ‘encouraged activity’ to an expectation. Practitioners noted that while organizational policies and contracts alone can be insufficient without ongoing monitoring, supervision, and support, they provide a clear foundation for accountability and long-term change:

Previously we had a very similar [healthy food retail] project… I don't think there was really any follow up or audit, so to speak. It was more so ‘hey, we want to try and do this’, compared to now being part of our food policy. I know it's brutal saying it, but the whole carrot and stick, there wasn't the big stick to go ‘hey, you're not in alignment with Council policy on this’. Previously it was just a ‘we're trying to influence some change’.

(Practitioner 6, sport and recreation, Victoria, regional)

Modifying physical infrastructure and equipment made healthier options easier to prepare, store, and promote to customers, while access to additional funding (e.g. small grants), enabled these infrastructure upgrades and product trials without fear of financial loss. Participants also described the need for ‘revising professional roles’ to integrate healthy food retail tasks into routine workflows and accountability structures (e.g. auditing tasks). Practitioners emphasized the importance of establishing or allocating sufficient time for a central (e.g. state-level) or organization-level (e.g. school) supporter role to provide ongoing oversight and technical assistance across outlets. Retailers offering house-made items also described how they have reconfigured staffing to support sustainment, such as shifting labour towards food preparation rather than customer-facing tasks to ensure that healthy food and drinks do not run out of stock.

We do have more staff preparing the food. But the way we've reconfigured our canteen, we only have either one, or if it's busy, two people on the till… We've got a scanner that scans everything, and we've made that process much faster, so we don't have lots of people having to sell the items.

(Retailer 5, sport and recreation, Queensland, metropolitan)

Lastly, network weaving and strategies involving multiple actors within and beyond organizations was considered central to sustainment. Practitioners highlighted the need for organizational consensus to support alignment across departments whose decisions may affect healthy food retail practices (e.g. contracts, finance, and procurement). Retailers emphasized broader food system coordination involving suppliers, manufacturers, governments, and consumers. Retailers and practitioners both commonly described the need for relationship-building with suppliers to improve access to suitable products. Network weaving and leadership support were also some of the most frequently mentioned unmet sustainment support needs, with most participants indicating that additional leadership support was required to bring key actors ‘on the same page’ to support long-term healthy food retail transformation. As one retailer and practitioner from the same governing organization reflected:

I think all the [local government-owned sporting] centres, we all need to be on the same page. I think we need to be… we need to be shown as united and as council facilities.

(Retailer 3, sport and recreation, Victoria, regional)

We very quickly realised that the long-term sustainability of this work [policy implementation] needed to be supported with refreshment of our policies and greater buy-in from leaders.

(Practitioner 7, sport and recreation, Victoria, regional)

## Discussion

This study explored how healthy food retail practices are sustained over time in Australian public foodservice settings, drawing on the perspectives of both retailers and practitioners. Retailers commonly described sustainment as feasible once practices were implemented and embedded into routine operations, whereas practitioners emphasized that sustainment was highly context-dependent and uneven across outlets. Participants described multiple factors influencing sustainment, with main themes relating to the fit between specific healthy food retail practices and food outlet context, the role of people and processes driving sustainment, and need for top-down leadership and systemic support. Participants also described multiple concurrent sustainment strategies aligned with these determinants, including iterative and evaluative strategies to support intervention fit, fidelity, and adaptation, building healthy food retail capacity and demand through engagement and behaviour-focused strategies, and embedding healthy food retail within organizational governance and policy systems.

These findings complement the emerging evidence on the sustainment of public health interventions, including healthy food retail initiatives. In this study, retailers generally perceived sustainment of healthy food retail practices, particularly changes to product availability, as achievable once successfully implemented. This contrasts with Law *et al*. (2021), who reported that retailers in Western Australian hospitals experienced mandatory policies as generating ongoing operational challenges that were difficult to meet without support. This difference may reflect variation in policy contexts, settings, and support available and how determinants were conceptualized and assessed. For example, Law *et al*. (2021) did not clearly distinguish between implementation and sustainment, and the short policy implementation timeframe may have intensified early implementation pressures, influencing retailers’ perceptions of long-term feasibility. Future research could compare food outlets that discontinued versus sustained healthy food retail practices following implementation to better understand variations in feasibility over time.

Consistent with implementation and sustainment theories (Shelton *et al*. 2018) and empirical evidence (Gupta *et al*. 2022), multi-level determinants were identified in this study, including the outer sociopolitical and food environment, inner contexts (governing organizations, and food outlets), and customer- and intervention-level factors. Our findings align with prior research highlighting the more prominent role of market and business dynamics in healthy food retail policy interventions (Blake *et al*. 2019, 2021) relative to other population-level health promotion interventions (Crane *et al*. 2022). While intervention costs, profitability, and customer appeal appear critical for sustainment, our findings extend on this by suggesting that these factors are closely tied to intervention-context fit. Specifically, sustained healthy food retail is likely to require flexibility and adaptability to identify available healthier products that are simultaneously policy-compliant, operationally feasible, and appealing to customers.

Given the context in which foodservice outlets operate, our findings suggest that sustainment is a dynamic process, requiring ongoing maintenance of intervention-context fit rather than rigid adherence to initially implemented practices (e.g. specific food items). This aligns with the Dynamic Sustainability Framework (Chambers *et al*. 2013) which emphasizes the need to sustain fit between interventions and changing organizational, policy, and market contexts through ongoing improvement and adaptation. Participants in this study described regular adaptation, for example through reviewing sales data and customer preferences, responding to rising food costs, and adjusting staffing to maintain healthy food retail practices over time.

Our findings also highlight sustainment as a multi-level and -actor process involving retailers, governing organizations, external partners, suppliers, manufacturers, governments, and customers, rather than something driven by short-term projects or food outlets alone. These findings align with system-level sustainment mechanisms of population-level health interventions described by Crane *et al*. (2022), which emphasize embedding practices within governance structures, organizational processes, and long-term resourcing. Moreover, evidence from Canadian convenience store contexts indicates that discontinuation is often driven by system-level constraints (e.g. food prices), rather than retailer motivation or resistance (Lynch *et al*. 2023). Our finding that governing organizations play a particularly important role in sustainment aligns with a recent policy analysis suggesting that Australian and New Zealand healthy food retail policies place primary responsibility for sustainment on governing organizations (Backman *et al*. 2026a), along with evidence from university healthy vending retail context identifying institutions as key change leaders (Dancey *et al*. 2025). In contexts where staff and customer support is already high, strengthening organizational leadership buy-in may be a key focus for future interventions (Rosin *et al*. 2024, Blake *et al*. 2025).

Our study identified multiple sustainment strategies from monitoring to consumer engagement, infrastructure changes, and governance mechanisms, involving multiple actors. These findings suggest that some degree of ‘*self-sustainment*’, defined as continuation of implemented practices without external implementation support (Wolfenden *et al*. 2024), may be achievable for low-burden healthy food retail practices that are commercially viable, widely accepted by customers, and easy to carry out with limited technical expertise (e.g. healthier drink availability). However, sustaining policy requirements perceived as more complex, higher-burden or misaligned with the business case (e.g. higher proportional availability of healthier foods relative to less healthy, high-margin foods) may be less feasible without ongoing oversight and support, indicating a need for both *static* (e.g. ongoing monitoring) and *dynamic* (e.g. tailored technical assistance) *sustainment supports* (Wolfenden *et al*. 2024). Many identified strategies align conceptually with the sustainment-explicit ERIC glossary (Nathan *et al*. 2022), suggesting similar sustainment mechanisms across health interventions, although some rewording and regrouping were required for the foodservice context. For example, multiple training-related ERIC strategies (e.g. conduct educational meetings, use train-the-trainer strategies) were regrouped as a single ‘train and continue upskilling the retailer and/or outlet staff’ strategy to better reflect the foodservice context (see Supplementary File S4 for details). Balis and Houghtaling (2023) have also previously adapted and regrouped ERIC strategies for community-based interventions; however, their focus remains on the implementation phase, with limited recognition of the business considerations that were identified as important in the current study.

### Implications for policy and practice

Our findings suggest that investment in practitioner and organizational capacity is important for sustaining healthy food retail sustainment support, given practitioners’ central role as ‘sustainment champions’ and governing organizations’ responsibility for oversight. Greater policy and monitoring consistency across jurisdictions and over time (Backman *et al*. 2026a), with flexibility in policy requirements and supplier engagement, may further support intervention-context fit and streamline support structures. Additionally, product availability requirements should be supported by policy strategies or actions that ensure the affordability and accessibility of healthier options to minimize the risk of exacerbating dietary inequalities.

Sustainment as a dynamic process means that food outlet practices are likely to fluctuate in response to policy updates, staffing changes, monitoring frequency, and leadership attention. This has potential implications for policy design, suggesting that infrequent government-prescribed monitoring, often every 1–3 years (Backman *et al*. 2026a), may be insufficient to prevent gradual drift or discontinuation of some practices. Greater investment in frequent and consistent monitoring and practical implementation tools, alongside resourcing practitioners to provide ongoing support to food outlets, could help strengthen sustainment efforts. Similar challenges have been reported in England in relation to the ‘Food (Promotion and Placement) (England) Regulations 2021’, where local authority officers responsible for enforcement have described limited capacity and a need for stronger support from policy setters (Dhuria *et al*. 2024).

### Strengths and limitations

This study has several strengths. It included both retailer and practitioner perspectives across multiple jurisdictions and settings in Australia, enabling identification of common sustainment experiences across roles and contexts. The use of established implementation science frameworks to guide data collection and analysis strengthened conceptual rigour, while analytic calibration through double coding helped enhance the credibility of interpretation. Another key strength of this study is that most participants (*n* = 12) had more than 2 years of sustainment experience, some with up to 17 years of experience. This suggests that the findings primarily reflect real-world experiences of sustained healthy food retail practices, rather than anticipated or shorter-term implementation experiences.

However, including five participants (29%) from settings with less than 2 years of sustainment experience is a limitation, as some findings may reflect shorter-term implementation rather than longer-term sustainment. Additionally, sustainment success was self-reported by participants and may be an over-reported due to social desirability bias. Future research could combine qualitative insights with objective measures of sustained practice (e.g. product availability assessments) and adopt longitudinal designs to assess the effectiveness of sustainment strategies and how sustainment determinants, support needs, and responsibilities evolve over time as organizational priorities, broader socio-political and food environments change. Lastly, the relatively small sample, which was dominated by participants representing sport and recreation settings, limited exploration of potential differences across settings, jurisdictions, and sustainment stages. While the study aimed to identify common determinants and strategies across public foodservice settings rather than compare contexts, future research could investigate how sustainment experiences vary between settings and stages of sustainment.

## Conclusion

Sustaining healthy food retail practices in public foodservice settings can be feasible but relies on alignment between outlet context, key actors, organizational governance and system-level support, and on maintaining this fit over time. Healthy food retail practices are most likely to be maintained when sustainment strategies are embedded within routine organizational systems and reinforced by supportive policy and broader food environments, and ongoing practical support. Clear and consistent policy guidance, sustained investment in practitioner roles, comprehensive and consistent monitoring systems, practical implementation tools, and stronger coordination with suppliers are needed to support long-term sustainment at scale. Future research should assess the effectiveness of sustainment strategies and how sustainment determinants, support needs, and experiences evolve over time across different public foodservice settings and stages of sustainment.

## Supplementary Material

daag092_Supplementary_Data

## Data Availability

The datasets cannot be shared publicly to protect the privacy of the people who participated in the study. Anonymized data can be shared on reasonable request to the corresponding author.
