# Supplementary material for: ‘We all need to be on the same page’: sustainment of healthy food retail practices in Australian public settings
Source: Health Promot Int. 2026 Jul 14;41(4):daag092. doi: 10.1093/heapro/daag092 (PMC13394710; doi:10.1093/heapro/daag092)
Supplement: daag092_Supplementary_Data [file daag092_supplementary_data.zip › HFR sustainment interviews_s-file 4 strategies final.docx]

### Supplementary File S4: Key healthy food retail sustainment strategies in public foodservice settings identified from interviews with implementation support practitioners (n=9) and retailers (n=8), mapped to the sustainment-explicit Expert Recommendations for Implementing Change (ERIC) glossary **^[[1]](#footnote-1)^**

| **Theme** | **Healthy food retail sustainment strategies relevant to this theme** | **Strategy description** | **Key actors** | **Corresponding ERIC strategies** |
| --- | --- | --- | --- | --- |
| **Use iterative, evaluative and adaptive strategies to support intervention fit and fidelity** | Monitor, provide feedback, and communicate the impact of healthy food retail practices | Routine monitoring and feedback processes used to assess alignment with healthy food retail requirements, support sustainment and continuous improvement, and assess impacts (e.g., customer purchasing patterns). This includes formal audits for compliance and reporting purposes (to governing organisations or governments), as well as retailer-led self-monitoring for internal quality assurance. Monitoring can also be used to reinforce expectations, incentivise compliance, and hold outlets accountable for agreed practices. | Governments, governing organisations, practitioners, retailers | Use evaluative and iterative strategies:   - Audit and provide feedback - Purposely reexamine the implementation - Communicate with stakeholders the continued impact of HFR practices |
|  | Develop and organise quality monitoring systems and tools | Establishing and maintaining structured systems, tools, and processes for monitoring of healthy food retail practices, including defining monitoring frequency and responsibility (e.g., retailer vs practitioner). This includes the development and introduction of standardised and low-burden assessment tools, reporting templates, and/or digital systems to streamline and support consistent monitoring. These systems, tools and processes are likely to evolve and require updates over time. | Governments, governing organisations, practitioners, health promotion organisations | Use evaluative and iterative strategies:   - Develop and organise quality monitoring systems - Develop and implement tools for quality monitoring   Change infrastructure:   - Change monitoring systems |
|  | Staged implementation and trialling to ensure contextual fit | An iterative approach to introducing healthier items (or other food retail practices) through small-scale trials to identify options that are acceptable to customers, operationally feasible, and commercially viable over time. Trialling can reduce the risk of introducing practices that cannot be sustained. | Retailers, practitioners | Use evaluative and iterative strategies:   - Conduct cyclical small tests of change - Stage implementation scale up |
|  | Obtain and use staff and consumer feedback | Ongoing collection and use of staff and consumer feedback to inform menu updates and assess the long-term suitability of healthier offerings. Feedback is used to refine practices, guide product selection, and support sustained customer demand. | Retailers, practitioners, consumers | Use evaluative and iterative strategies:   - Obtain and use consumer feedback - Conduct local needs assessment |
|  | Tailor healthy food retail practices and promote adaptability | Prioritising healthy food retail practices that fit local capacity and resources, including adapting menu changes and strategies to minimise workload and cost. Tailoring can be used to enhance feasibility and reduce burden, particularly in low-resource or volunteer-run settings. | Practitioners, (retailers) | Provide interactive assistance:   - Tailor strategies - Provide local technical assistance - Promote adaptability |
| **Building healthy food retail capacity and demand through engagement and behaviour-focused strategies** | Train and continue upskilling the retailer and/or outlet staff | Ongoing, practical training to build retailer and staff capability to implement and sustain healthy food retail practices, particularly where tasks may be more complex (e.g., food classification, compliance monitoring) and staff turnover is common. Training focused on building confidence, understanding the rationale for changes (“the why”), and developing skills to apply classification systems (e.g., traffic lights) and interpret policy guidance independently. Embedding training into staff induction and routine professional development trainings. | Practitioners, governing organisations, retailers,  outlet staff | Train and educate stakeholders:   - Conduct educational meetings - Conduct educational outreach visits - Conduct ongoing training - Use train-the-trainer strategies |
|  | Develop and distribute educational and practical implementation support materials | Provision of standardised, easy-to-use tools and resources to reduce cognitive and time burden on outlet staff and minimise reliance on ongoing external support. This included classification tools, planograms, recipe handbooks, documented procedures, and point-of-sale-integrated prompts (e.g., traffic light colour-coded buttons). Such materials can support consistency across sites, guide restocking and food preparation, and help maintain healthy food retail practices despite staff turnover and operational change. Educational materials can also be used for customer-facing communication to explain changes and reinforce healthier norms. | Practitioners, governing organisations, health promotion organisations, governments | Train and educate stakeholders:   - Distribute educational materials - Review and update educational materials   Support clinicians (retailers):   - Remind clinicians (retailers)   Change physical structure and equipment:   - Change physical structure and equipment |
|  | Provide ongoing tailored local supervision and technical assistance | Responsive and locally tailored support to assist outlets with technical tasks such as food classification, menu changes, sourcing suitable products, pricing decisions, in-store marketing, and compliance processes. Support intensity can vary by outlet capacity and can be more important in settings with high staff turnover, volunteer staff, or limited nutrition expertise. | Practitioners, governing organisations | Provide interactive assistance:   - Provide clinical (food outlet) supervision - Provide local technical assistance - Tailor strategies - Promote adaptability - Facilitation - Provide ongoing consultation |
|  | Provide centralised technical assistance | Centralised technical assistance refers to the provision of dedicated implementation and sustainment support through state-, local-, or project-level roles (e.g., dietitians or health promotion officers) whose primary role is to support multiple food outlets. This higher-level support includes guidance on product classification, menu changes, merchandising, pricing, and compliance processes, as well as troubleshooting and coordination across outlets to reduce duplication and variation in practice. Scalable implementation support tools (e.g., FoodChecker or similar food classification and policy compliance assessment tools) can also be as a form of centralised support. | Retailers, governing organisations, health promotion organisations, governments | Provide interactive assistance:   - Centralise technical assistance   Train and educate stakeholders:   - Distribute educational materials |
|  | Involve customers in menu and product offering decisions | Actively involving customers in menu and product planning (e.g., through surveys, informal feedback, and taste testing) can guide decisions about which healthier items to introduce or retain. Consumer involvement can help build acceptability, reduce perceived financial risk for outlets, and improve long-term fit by identifying options that were both appealing and financially viable. | Retailers, practitioners, governing organisations, customers | Engage consumers:   - Involve consumers and family members - Prepare consumers to be active participants   Use evaluative and iterative strategies:   - Obtain and use consumer feedback - Conduct local needs assessment |
|  | Utilise marketing strategies to increase and sustain demand for healthier options | Using marketing strategies to sustain demand by making healthier options easy, appealing, and financially sensible choices. This can include ongoing promotion and communication (e.g., signage, tailored communication materials) and improving convenience and visual appeal (e.g., “grab-and-go” formats, attractive displays). Pricing strategies can be used to increase demand and support financial viability. | Retailers, practitioners, governing organisations, customers | Engage consumers:   - Increase demand - Use mass media   Utilise financial strategies   - Alter consumer fees |
| **Embedding healthy food retail within organisational governance and policy systems** | Involve executive boards and organisational leaders | Engaging executive boards and senior organisational leaders to endorse healthy food retail priorities and provide ongoing oversight. Leadership involvement is important for setting clear expectations, maintaining healthy food retail on organisational agendas, and ensuring consistency across departments (e.g., procurement, leasing, budgets, education, and compliance). | Governments, governing organisations,  health promotion organisations, practitioners | Develop stakeholder interrelationships:   - Involve executive boards - Re-engage with local opinion leaders |
|  | Promote network weaving | Building and maintaining relationships within organisations and between key interest-holders to support sustainment of healthy food retail practices. This can include coordination across internal departments, as well as engagement with external actors such as suppliers, manufacturers, other retailers, customers, and government agencies. Network weaving can support aligning product supply with policy requirements, reinforcing shared expectations, enabling peer learning, and supporting consistent health-promoting messages across the food system. | All | Develop stakeholder interrelationships:   - Promote network weaving - Build a coalition |
|  | Revise professional roles to embed healthy food retail and sustainment activities | Embedding healthy food retail responsibilities into specific roles (within governing organisations and/or food outlets) and workflows, rather than relying on ad hoc or project-based effort. Clarifying “who does what”, how often, and through which systems. This can include assigning clear responsibility for monitoring and follow-up to dedicated positions or teams (e.g., integrating routine compliance checks into existing practitioner roles), embedding food classification and compliance controls into leadership roles (e.g., executive, board or manager approval when introducing new items on the menu), or reconfiguring staffing models and task allocation to better support healthy food retail operations (e.g., shifting labour towards food preparation). | Governing organisations, practitioners, retailers | Support clinicians (retailers):   - Revise professional roles |
|  | Appoint for sustainment leadership and champions | Appointment of one or more leaders or champions who coordinate sustainment activities and reinforce expectations over time. Effective champions have dedicated time, relevant nutrition or implementation expertise, and ideally sufficient authority to work across relevant teams (e.g., procurement, facilities, retail staff). However, leadership can be shared across roles or teams rather than relying on a single individual, reducing the risk of drift when staff change. | Practitioners, governing organisations, retailers | Develop stakeholder interrelationships:   - Identify and prepare champions - Recruit, designate, and train for leadership |
|  | Strengthen and formalise healthy food retail compliance requirements through governance and contracts | Shifting healthy food retail from voluntary or encouraged practice to formalised requirements. Embedding healthy food retail expectations into government policy mandates, organisational policies, contracts, lease agreements, and documented procedures to shift practices from voluntary or project-based activities to routine business operations. Formalisation includes clear and consistent requirements across policy and organisational levels, alignment across departments, and integration into operational systems (e.g., procurement rules, handover processes, standardised tools). This approach reduces reliance on individual motivation, supports continuity during staff turnover, and helps prevent drift when priorities or staff change. | Governments, governing organisations (including different departments) | Change infrastructure:   - Change liability laws - Mandate change   Utilise financial strategies   - Fund and contract for the clinical innovation (healthy food retail practices) |
|  | Change physical structure, equipment and systems | Change physical layouts, equipment, and digital systems to make healthier options easier to prepare, display, and sell by default. Examples include reconfiguring fridges and product displays, investing in equipment for more effective healthy food preparation, and embedding classification prompts or reporting dashboards into POS systems. These changes can reduce workload and reliance on staff knowledge, and improve convenience and appeal. | Retailers, governing organisations, practitioners | Change infrastructure:   - Change physical structure and equipment |
|  | Access and use funding to support sustainment | Access to external funding (e.g., grants, equipment subsidies, funded support roles) can help reduce financial risk and enable outlets to make infrastructure and system changes that support long-term sustainment. For example, funding can be used for equipment purchases, menu trials and taste testing. | Governments, governing organisations, practitioners,  retailers | Utilise financial strategies:   - Access new funding |

1. Sustainment-explicit Expert Recommendations for Implementing Change by Nathan et al. (2022) offers a comprehensive glossary of strategies to support adoption, implementation and sustainment, categorised by these three different phases.

   *Nathan, N., Powell, B. J., Shelton, R. C., Laur, C. V., Wolfenden, L., Hailemariam, M., Yoong, S. L., Sutherland, R., Kingsland, M., Waltz, T. J., & Hall, A. (2022). Do the Expert Recommendations for Implementing Change (ERIC) strategies adequately address sustainment? [Original Research]. Frontiers in Health Services, 2.* [*https://doi.org/10.3389/frhs.2022.905909*](https://doi.org/10.3389/frhs.2022.905909) [↑](#footnote-ref-1)
