# Supplementary material for: ‘We all need to be on the same page’: sustainment of healthy food retail practices in Australian public settings
Source: Health Promot Int. 2026 Jul 14;41(4):daag092. doi: 10.1093/heapro/daag092 (PMC13394710; doi:10.1093/heapro/daag092)
Supplement: daag092_Supplementary_Data [file daag092_supplementary_data.zip › HFR sustainment interviews_supplementary file 1.docx]

### Supplementary File S1: Recruitment and eligibility screening instrument for qualitative interviews exploring sustainment of healthy food retail practices in Australian public settings


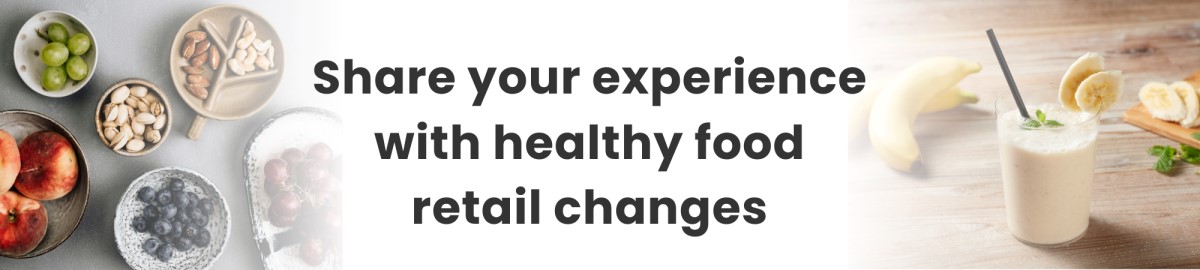


Photos: Canva

Deakin University is conducting research to understand what makes it **easier or harder** for food outlets to **make healthy food retail changes**. Your insights can help shape better strategies to support food businesses.

Your involvement in this research includes a **45-60minute interview**, which can be conducted either in person or via Zoom or MS Teams. Please review the **Plain Language Statement** on the next page for more details about the study before agreeing to participate.

As a token of appreciation for your time, you will receive a **$100 Prezzee eGift card** upon completing the interview

Tick the box below to continue


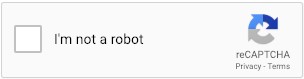


Thank you for your interest in this research project. Please answer **a few quick questions** below to determine your eligibility. Your responses are confidential and used only for screening purposes.

Are you based in Australia, aged over 18 years, able to provide informed consent, and are able to read, speak and understand English?

Yes

No

Do you own, manage or work in a kiosk, café, canteen, or another food outlet that primarily offers food for immediate consumption?

Yes

No

Are you a person whose role involves supporting food outlets that primarily offer food for immediate consumption in making healthy retail changes?

Yes

No

Do you have experience making healthier food and drink options available in food outlet(s) based on government policies/guidelines?*

*E.g., [Victorian Healthy Choices guidelines,](https://www.health.vic.gov.au/preventive-health/healthy-choices) [Smart Choices Healthy Food and Drink Supply Strategy for Queensland](http://education.qld.gov.au/student/Documents/smart-choices-strategy.pdf), [Schools](http://education.qld.gov.au/student/Documents/smart-choices-strategy.pdf), [Australian guide to healthy eating | Eat For Health,](https://www.eatforhealth.gov.au/guidelines/australian-guide-healthy-eating) [WA School Food and Drink Criteria for WA schools](https://www.freshsnap.org.au/resource/a-fresh-nutrient-criteria-for-wa-schools/))

Yes

No

No, but I have experience making other healthy changes (please describe what)

Unsure (please describe why unsure)

Have you been involved in maintaining or attempting to maintain* any healthier food and drink changes in food outlets for at least 2 years?

*The changes don’t need to have lasted for 2 years, but they must have first been made 2 or more years ago

Yes, I have been involved for 2 years or more

No, I have been involved for less than 2 years (please specify for how long)

No, I have not been involved in maintaining these changes

Which best describes your food outlet’s/outlets' setting(s)? Select all that apply

School (primary or secondary)

Healthcare or hospital

Sport and recreation (e.g., aquatic centre)

Library, theatre, museum or other cultural setting

University

Workplace

Other (please specify)

**Congratulations – based on the initial screening, you appear to be eligible to participate in this study!**

Before agreeing to participate, please read the **Plain Language Statement** below. It contains important information about the research project, what your participation involves, and your rights as a participant.

Click the link below to view the statement. You can also download a copy for your records:

[**Plain Langua**](https://researchsurveys.deakin.edu.au/CP/File.php?F=F_b2El0QGtFAEvVZk)**g**[**e Statement**](https://researchsurveys.deakin.edu.au/CP/File.php?F=F_b2El0QGtFAEvVZk)

To continue, select one of the below:

Yes, I have read the Plain Language Statement and **AGREE** to participate in this research

No, I **DO NOT agree** to participate in this research

Please provide your contact details so researchers can arrange a time for an interview

Your name

Email address

Phone number (optional)

Would you prefer an interview in-person or online (via Zoom or MS Teams)? Select all that apply

In-person (participants based in Victoria)

Zoom

Microsoft Teams

Provide three dates and times you are available for a **45-60-minute interview** within the following timeframe:

Monday - Friday

May - September 2025

Date

Time

Time zone

(

e.g. AEST/Melbourne time

)

Option 1

Option 2

Option 3

[Powered by Qualtrics](https://www.qualtrics.com/powered-by-qualtrics/?utm_source=internal%2Binitiatives&utm_medium=survey%2Bpowered%2Bby%2Bqualtrics&utm_content=%7b~BrandID~%7d&utm_survey_id=%7b~SurveyID~%7d)

### 
