# Supplementary material for: ‘We all need to be on the same page’: sustainment of healthy food retail practices in Australian public settings
Source: Health Promot Int. 2026 Jul 14;41(4):daag092. doi: 10.1093/heapro/daag092 (PMC13394710; doi:10.1093/heapro/daag092)
Supplement: daag092_Supplementary_Data [file daag092_supplementary_data.zip › HFR sustainment interviews_supplementary file 2 final.docx]

### Supplementary File S2: Semi-structured interview guide used to explore retailer and implementation support practitioner experiences of sustaining healthy food retail practices in Australian public settings

| Questions | Prompts | Who | Targeted construct |
| --- | --- | --- | --- |
| Can you briefly introduce yourself and your role? |  | All participants | Participant background |
| Can you briefly describe the type of food outlet you work in and the communities it serves? | Setting, outlet type/size, typical product offering, customer demographics | Retailers | Outlet context |
| Can you briefly describe the type(s) of food outlet(s) you work with and the communities they serve? |  | Practitioners |  |
| How long have you been working in this role? |  | All participants | Participant background |
| Has your outlet implemented or used any government policies or guidelines related to healthier food offerings? |  | Retailers | Policy context |
| Can you briefly describe the government policies related to healthier food offerings relevant to the settings you work with? |  | Practitioners |  |
| Can you describe the changes in the food outlet product offerings or marketing practices you have made? | Product offerings, placement, pricing, promotions | Retailers | Healthy food retail practices |
| Can you describe the changes in the food outlet product offerings or marketing practices you have been involved implementing? |  | Practitioners |  |
| When was the first time these changes were introduced at your outlet? |  | Retailers | Implementation and sustainment timeline |
| When was the first time these changes were introduced at the outlets you work with? |  | Practitioners |  |
| What has been your involvement in implementing or sustaining these changes? | Level of responsibility, day-to-day activities | All participants | Personal role in sustainment |
| What has been your overall experience in sustaining these changes / healthy food retail practices over time? | Easy or challenging, changes have been maintained or not maintained | All participants | Overall feasibility of sustainment |
| What factors have made it easier to sustain these changes? Have there been any changes that have been easier to sustain than others? | Outer contextual, inner contextual, processes, people | All participants | Facilitators to sustainment |
| What factors have made it harder to sustain these changes? Have there been any changes that have been more difficult to sustain than others? |  |  | Barriers to sustainment |
| How have you managed these (challenges)? |  | Retailers | Sustainment strategies and support |
| What strategies have you used to support long-term changes? |  | Practitioners |  |
| Have you received any support to address these challenges related to keeping changes in place (e.g., from government, health-promoting organisations)? If so, what has been most useful (if anything)? |  | All participants |  |
| Based on your experience, what strategies do you think are most effective in supporting food outlets to sustain these changes / healthy food retail practices over time? |  | All participants |  |
