# Supplementary material for: ‘We all need to be on the same page’: sustainment of healthy food retail practices in Australian public settings
Source: Health Promot Int. 2026 Jul 14;41(4):daag092. doi: 10.1093/heapro/daag092 (PMC13394710; doi:10.1093/heapro/daag092)
Supplement: daag092_Supplementary_Data [file daag092_supplementary_data.zip › HFR sustainment interviews_supplementary file 3 final.docx]

### Supplementary File S3: Multi-theory coding framework used to code interviews with implementation support practitioners (n=9) and retailers (n=8) to explore determinants and strategies influencing the sustainment of healthy food retail practices in Australian public settings

| **Domain** | | **Parent code** | **Descriptive codes** | **Code description** | **Theoretical framework / evidence** |
| --- | --- | --- | --- | --- | --- |
| **Context** | | | | | |
|  | Participants | Participant role | Retailer  Manager  Staff (full time or part time)  Staff (casual)  Staff (volunteer)  Dietitian  Health promotion practitioner  Other (inductively coded) |  |  |
|  | Policy context | Policies relevant to the setting or food outlet | Inductively coded (semantic) |  |  |
|  | Food outlet context | Outlet setting | Healthcare  School  Sport and recreation  Other (inductively coded) |  | Priority settings based on available policies (Backman et al., 2026) |
|  |  | Product offerings | Commercially packaged snacks  Commercially pre-prepared meals  Commercially packaged drinks  Alcoholic drinks  Made-to-order food  Made-to-order drinks  Ready-to-eat foods  Vending machines  Other (inductively coded) | Commercially packaged snacks (e.g., confectionery, nut mixes, ice cream- products purchased from suppliers and sold as-is)  Commercially pre-prepared meals (e.g., pre-packaged sandwiches, wraps, pies, heat-and-eat meals- products purchased from suppliers)  Commercially packaged drinks (e.g., bottled or canned soft drinks, juices, flavoured milk)  Alcoholic drinks (bottled/canned, from tap, mixed onsite)  Made-to-order food (e.g., meals freshly prepared upon customer request)  Made-to-order drinks (e.g., coffee, smoothies, milkshakes- freshly prepared beverages)  Ready-to-eat foods prepared in-house (e.g., sandwiches, wraps, salads, cakes, slices- prepared on-site but sold pre-made for quick selection)  Vending machines (e.g. Drinks, snacks or both) |  |
|  |  | Outlet size (described by participants) | Inductively coded |  |  |
|  | Healthy food retail experience | Healthy food retail practices implemented | Changes in:  Food availability (healthy)  Food availability (unhealthy)  Drink availability (healthy)  Drink availability (unhealthy)  Placement changes  Pricing changes  Promotional changes  Other (inductively coded) |  | Marketing mix (Goi, 2009) |
|  | Sustainment timeframe | Initial implementation | First experience with healthy food retail practices: 2 years  First experience with healthy food retail practices: 2-4 years  First experience with healthy food retail practices: 4-6 years  First experience with healthy food retail practices: 6 or more years  Other | Participants implemented healthy food retail practices in food outlet(s) for the first time x years ago |  |
|  | Sustainment feasibility | Overall perceived feasibility to sustain healthy food retail practices | Easy  Some aspects easy, some difficult  Difficult  Other (inductively coded) | How easy/difficult it has been to maintain healthy practices over time (overall experience) |  |
|  |  | Components easer to sustain  (if mentioned) | Food availability (healthy)  Food availability (unhealthy)  Drink availability (healthy)  Drink availability (unhealthy)  Placement practices  Pricing practices  Promotional practices  Other | Which types of healthy food retail practices are easy to sustain from participant perspective (if mentioned) | Marketing mix (Goi, 2009) |
|  |  | Components more difficult to sustain  (if mentioned) |  | Which types of healthy food retail practices are difficult to sustain from participant perspective (if mentioned) |  |
| **Determinants of sustainment: barriers and enablers** | | | | | |
|  | Barrier  Enabler | | All determinants coded as ‘barriers’ and ‘enablers’ first before coding them against specific determinant domains | | |
|  | Outer contextual | | Sociopolitical environment (including policies and legislation) | The broader sociopolitical context, including existing policies, regulations, guidelines, and mandates that may impact the sustainment of healthy food retail practices. | Integrated Sustainability Framework (ISF) (Shelton et al., 2018) – adapted to food retail ^[[1]](#footnote-1)^ |
|  |  |  | Broader food environment (e.g., food market, food marketing, weather) | Broader food environment factors such as natural resources, weather conditions, and agricultural subsidies, which can impact the market supply and general availability of healthy items. This includes the appeal of healthy options outside of food retail outlets, shaped by promotional methods used by stakeholders, including marketing strategies by food manufacturers. |  |
|  |  |  | External recognition for healthy food retail practice | External recognition for healthy food retail changes made (e.g., accreditation/certification programs, media attention, etc.) |  |
|  |  |  | National / governmental leadership | Government leading healthy food retail initiatives (e.g., government-led policy actions that target food retail practice). |  |
|  |  |  | Community ownership | Members of the community leading efforts to promote healthy food retail practices. |  |
|  |  |  | Values, priorities and needs of the broader population | Healthy food retail practice is regarded as a national priority or fits with national, state, or local government priorities, needs, and values. For example, governmental prioritisation of healthy food retail transformation to prevent diet-related diseases. |  |
|  |  |  | Government funding environment | The allocation of government budgets and the overall funding environment either support or hinder healthy food retail practices. |  |
|  |  |  | Other (inductively coded; semantic and latent) |  |  |
|  | Interorganisational factors | | External partners, support and resources | Support and resources provided by external partners, such as local governments, health departments, health promotion agencies, universities and other supportive organisations. This includes non-financial resources provided by these stakeholders. |  |
|  |  |  | Food supply and supplier related factors | Characteristics of food distributors/suppliers and availability, accessibility and affordability of healthy food options provided by distributors/suppliers to retailers. |  |
|  |  |  | Competition | Availability of other food outlets, their product offerings, and their locations, which may create competition among food outlets. |  |
|  |  |  | External funding | Funding and financial support provided by external stakeholders or governing organisations (e.g., health departments, health promotion agencies, local governments/councils, other governing organisations) to support implementation and/or sustainment of healthy food retail practices. |  |
|  |  |  | Other (inductively coded; semantic and latent) |  |  |
|  | Characteristics of customers | | Customer motivation and purchasing behaviour | General healthiness of customer purchases and customers’ response to healthy changes in the food retail outlet. |  |
|  |  |  | Customer characteristics | Customer demographics, socioeconomic status etc. |  |
|  |  |  | Customer needs | Perceived or actual need for healthier food options within a community. |  |
|  |  |  | Other (inductively coded; semantic and latent) |  |  |
|  | Inner context (food outlet and governing organisation) | | Role of governing organisations (leadership) | Governing organisations (e.g., local governments that own or oversee food outlets in public settings) driving the normalisation of healthy food retail practices. [capacity, motivation etc] |  |
|  |  |  | Characteristics of the food outlet (business)* | This includes factors such as the type of food outlet (e.g., supermarket, small store, cafe), its location, branding or organisational identity (e.g., whether it identifies as a "healthy food outlet"), and its organisational structure (e.g., independently owned vs. Part of a larger chain, number of employees, etc.). |  |
|  |  |  | Material resources | Availability and condition of material resources required to sustain the healthy food retail practices. This includes appropriate equipment such as operational refrigerators for storing fresh, healthy foods. |  |
|  |  |  | Internal funding and financial resources | Internal funding available and/or allocated for implementing and sustaining healthy changes in the product offering. |  |
|  |  |  | Organisational culture and climate | General organisational climate/culture is supportive of healthy food retail practice. |  |
|  |  |  | Capacity (human resources and time) | Availability of human resources and time required to sustain healthy changes in food outlets. For example, availability of staff with the relevant nutritional knowledge/experience needed to identify healthy and less healthy foods. |  |
|  |  |  | Mission and internal policies (alignment with healthy practices) | Healthy food retail practices are aligned with the organisation's mission, strategies, plans, and policies. For example, promoting healthy food retail practices is a priority within the organisation's overarching goals and objectives. |  |
|  |  |  | Organisational stability / staffing turnover | Stability of the organisation, including staff attrition and turnover rates, changes in leadership, and organisational restructuring, which can all influence the sustainment of healthy food retail practices. |  |
|  |  |  | Champions | Food retail organisations or individuals that have strong influences on other retailers or food outlet staff and promote the ongoing delivery of healthy food retail practices. |  |
|  |  |  | Other (inductively coded; semantic and latent) |  |  |
|  | Characteristics of the implementation support practitioner | | Practitioner characteristics, motivation and attitudes | Refers to the implementation support practitioner’s values, beliefs, motivation towards supporting healthy food retail initiatives. |  |
|  |  |  | Practitioner knowledge, skills and expertise | Refers to practitioner’s knowledge, skills and practical experience relevant to supporting and sustaining healthy food retail practices. |  |
|  | Characteristics of the retailer and staff | | Retailer and/or store manager characteristics, motivation and attitudes | Retailer and/or store manager's motivation and willingness to sustain healthy practices. For example, store manager’s support and perceived risks and benefits of the healthy food retail intervention. This can be influenced by retailer's/ manager's previous (successful or unsuccessful) experience with healthy food initiatives. |  |
|  |  |  | Food outlet staff characteristics, motivation and attitudes | Food outlet staff's motivation and willingness to sustain healthy changes. |  |
|  |  |  | Retailer and/or store manager knowledge, skills and expertise | Retailer/store manager's skills and knowledge required to sustain healthy food retail practice. For example, if a retailer has significant experience in successfully implementing and sustaining healthy food retail initiatives, it suggests that they have likely developed expertise in this area. |  |
|  |  |  | Food outlet staff’s knowledge, skills and expertise | Food outlet staff's skills and knowledge required to implement and sustain healthy food retail practice, including previous experience with healthy food retail interventions. |  |
|  |  |  | Other (inductively coded; semantic and latent) |  |  |
|  | Characteristics of the intervention (=healthy food retail practices) | | Profitability and cost | Perceived and actual financial benefits and costs associated with maintaining healthy changes in food retail outlet (e.g., new revenue stream, food waste from fresh food). |  |
|  |  |  | Burden and complexity | Retail outlet staff, manager, and/or owner time and resources required to implement and sustain healthy changes in the food outlet. This includes considering the complexity of the intervention components, workload and financial investments required from staff, managers, or owners to support and sustain these practices over time. |  |
|  |  |  | Fit with population and context | Fit of a healthy food intervention within a context, populations (customers), and retail organisations. This refers to how well the intervention aligns with the specific characteristics and needs of the community, the target customer base, and the operational structure of the retail organisations. |  |
|  |  |  | Intervention components * | The individual elements or strategies within an intervention that may vary in their ease of sustainment. Some components might be simpler to maintain over time due to factors like lower resource demands, higher perceived value, or ease of integration into existing systems, while others might require more effort, support, or resources to sustain effectively. |  |
|  |  |  | Adaptability | Degree to which a healthy food retail intervention can be tailored or refined to fit different settings or population needs. |  |
|  |  |  | Influence on population health | Perceived and actual influence/benefits of the healthy changes in food retail outlet on population health outcomes. |  |
|  |  |  | Trialability | Ease with which a healthy food retail intervention can be tested on a smaller scale before full implementation. |  |
|  |  |  | Other (inductively coded; semantic and latent) |  |  |
|  | Processes | | Fidelity, monitoring and enforcement | Processes aimed at maintaining fidelity to intervention components (e.g., through monitoring and auditing) and ensuring accountability of food outlets. |  |
|  |  |  | Partnership and engagement | Establishing and maintaining partnerships with key stakeholders, such as local governments, health departments, health promotion agencies, universities and other supportive organisations, to support and sustain healthy practices. This includes ongoing engagement with key stakeholders. |  |
|  |  |  | Technical assistance, support and supervision | Processes employed by external stakeholders to offer technical support and/or supervision for sustaining healthy food retail practices in food outlets. This may involve providing practical support (e.g. Updating lists of available healthy product options) as well as regular contact and site visits. |  |
|  |  |  | Increasing or creating consumer demand for healthy options | Processes employed by the retailer or external stakeholders to increase or create demand for healthy foods and drinks. For example, improving the appeal of the store and promotion of healthy food and drinks. |  |
|  |  |  | Communication | Internal communication: processes to ensure that retail outlet staff are informed about the initiative and new practices.  External communication: communication between retailers and stakeholders supporting the initiative. |  |
|  |  |  | Training | Processes that can influence retail manager and staff knowledge, skills, and motivation to implement and sustain healthy practices in the retail outlet. |  |
|  |  |  | Capacity building | Activities/processes that are aimed at empowering food retailers to sustain healthy food retail practices over time. These activities aim to provide retailers with the necessary skills, knowledge, and resources to continue offering healthy food options effectively. |  |
|  |  |  | Planning | Ongoing sustainment of the healthy food retail practices is planned. |  |
|  |  |  | Adaptation | Processes to actively and systematically guide adaptation of a healthy food retail initiative to better fit the target food outlets. |  |
|  |  |  | Evaluation and evidence: effectiveness | Collecting and presenting evidence to guide the planning and decision-making processes for healthy food retail interventions. For example, using available evidence to develop intervention strategies that are grounded in research and likely to result in sustained changes. Additionally, this includes offering evidence-based feedback to food outlets to illustrate the effects of the healthy practices implemented in their product offerings. |  |
|  |  |  | Team/board functioning | Sustainment processes are integrated as part of existing food outlet practices. |  |
|  |  |  | Contractual obligations / agreements | Healthy food retail practices are required as part of long-term rental contracts or other agreements with retailers in public settings. Also refers to retailer-supplier contracts. |  |
|  |  |  | Rapport building | Refers to the process of establishing and maintaining a trusting, respectful and collaborative relationship between the implementation support practitioner and the retailer or food outlet staff, including mutual understanding to support ongoing sustainment of healthy food retail practices. |  |
|  |  |  | Other (inductively coded; semantic and latent) |  |  |
| **Sustainment strategies and support needs** | | | | | |
|  | Use evaluative and iterative strategies | | Audit and provide feedback | Regularly collect and review data on the healthiness of products, menus, and/or in-store promotional activity in food outlets. Provide this feedback to managers, staff, and/or supporting organisations to guide decisions, monitor progress, and make improvements. | Expert Recommendations for Implementing Change (ERIC) (sustainment) (Nathan et al., 2022)^[[2]](#footnote-2)^ |
|  |  |  | Communicate with stakeholders the continued impact of healthy food retail practices | Share results and evidence with key actors (e.g., outlet managers, staff, customers, and local government partners) to demonstrate the ongoing benefits of healthy retail practices, such as improved customer satisfaction, community health outcomes, or financial performance. |  |
|  |  |  | Conduct cyclical small tests of change | Introduce healthy retail practices in small, incremental steps (e.g., adding a healthier product, adjusting placement, or trialling a price change), monitor their impact, and refine approaches before scaling up. This process continues over time and refinement is added at each cycle (i.e. Continuous improvement) |  |
|  |  |  | Conduct local needs assessment | Collect and analyse data related to the initial and ongoing need for and fit of the intervention (specific healthy food retail practices). |  |
|  |  |  | Develop a formal implementation blueprint | Create a clear, written plan outlining goals, responsibilities, timelines, and performance measures for implementing and sustaining healthy food retail practices. Update the plan as needed to ensure long-term sustainment. |  |
|  |  |  | Stage implementation scale up | Introduce healthy food retail practices gradually, starting with small pilots or demonstration projects, and expand over time to additional menu categories, outlets, or settings. |  |
|  |  |  | Develop and implement tools for quality monitoring | Develop, test, and introduce practical tools (e.g., checklists, scorecards, digital apps) to monitor the healthy food retail practices. |  |
|  |  |  | Develop and organise quality monitoring systems | Develop and organise systems and processes (e.g., regular reporting requirements, data dashboards) that track and review outlet compliance with healthy food retail standards/best practices or guidelines. |  |
|  |  |  | Obtain and use consumers  (and family) feedback | Collect customer feedback on healthy food retail changes (e.g., satisfaction, purchasing behaviours, preferences) and use this information to adapt and improve healthy food retail practices. |  |
|  |  |  | Purposely reexamine the  Implementation | Monitor progress and adjust healthy food retail practices and implementation strategies to continuously improve the food outlet practices (from health promotion perspective). |  |
|  |  |  | Other (inductively coded; semantic) |  |  |
|  | Provide interactive assistance | | Centralise technical assistance | Develop and use a centralised system to provide technical support focused on implementing and sustaining healthy food retail practices. |  |
|  |  |  | Facilitation | A process of interactive problem-solving and support to help outlets address challenges and strengthen implementation and sustainment through a supportive interpersonal relationship. |  |
|  |  |  | Provide food outlet supervision | Provide ongoing supervision and guidance for outlet staff responsible for healthy food retail practices. |  |
|  |  |  | Provide local technical assistance | Deliver technical support locally to outlets, using staff or partners who understand the local context and sustainment issues. |  |
|  |  |  | Promote adaptability (over time) | Identify how healthy food retail practices can be tailored to meet local needs while maintaining core elements. Continue to adapt strategies to ensure they remain appropriate, relevant, and sustainable over time. |  |
|  |  |  | Tailor strategies | Adapt implementation or sustainment strategies to address outlet-specific barriers and build on facilitators identified through ongoing data collection and feedback. |  |
|  |  |  | Use data experts | Involve, hire, and/or consult experts to guide outlets and supporting organisations in using data to monitor and improve implementation and sustainment of retail practices. |  |
|  |  |  | Use data warehousing techniques | Integrate and share monitoring data across outlets or organisations (e.g., from multiple outlets or settings) to inform sustainment efforts and support decision-making. |  |
|  |  |  | Other (inductively coded; semantic) |  |  |
|  | Develop stakeholder interrelationships | | Build a coalition | Recruit, cultivate, and maintain relationships with partners (e.g., local governments, health services, community organisations, health-promoting organisations) to support the implementation and sustainment of healthy food retail practices. |  |
|  |  |  | Capture and share local knowledge | Document and share experiences from outlets and implementers about what has worked in practice, so others can learn and adapt these strategies. |  |
|  |  |  | Conduct local consensus  Discussions | Engage local stakeholders (e.g., managers, suppliers, community representatives) in discussions to agree on priorities, ensure relevance, and strengthen buy-in for healthy food retail initiatives. |  |
|  |  |  | Involve executive boards | Engage leadership structures (e.g., boards, councils, or senior management) in overseeing and supporting implementation and sustainment, including reviewing progress and providing direction. |  |
|  |  |  | Re-affirm formal commitments | Review and reinforce written commitments made by partners to sustain healthy food retail practices. Assess whether additional commitments are needed to facilitate sustainment. |  |
|  |  |  | Promote network weaving | Identify, build and strengthen networks and working relationships within and across organisations (e.g., local councils, retailers, health promotion agencies) to encourage information sharing, collaborative problem-solving, and joint sustainment efforts. |  |
|  |  |  | Use advisory boards and  Workgroups | Establish advisory groups or working groups of key stakeholders to provide input, guidance, and feedback on implementing and sustaining healthy retail practices. |  |
|  |  |  | Develop academic partnerships | Partner with universities or researchers to support training, evaluation, and ongoing improvements in the implementation and sustainment of healthy food retail practices. |  |
|  |  |  | Identify successful sustainers | Identify food outlets or retailers who have successfully sustained healthy food retail practices and learn from their experiences to guide others. |  |
|  |  |  | Re-engage with local opinion leaders | Periodically re-engage with influential local leaders (e.g., community leaders, senior health promotion staff) about the importance of continuing healthy food retail practices, so they can influence others to maintain these practices. |  |
|  |  |  | Identify and prepare champions | Identify and support individuals who dedicate themselves to driving healthy food retail practices, and who can overcome resistance or indifference within their outlet or setting. |  |
|  |  |  | Organise implementation team meetings | Develop and support teams within outlets (or across multiple outlets/organisations) who are implementing healthy retail practices, providing time to reflect, share lessons learned, and support one another’s efforts. |  |
|  |  |  | Recruit, designate, and train for leadership | Recruit, designate, train and retrain as necessary, leaders or coordinators for the healthy food retail change |  |
|  |  |  | Use an implementation advisor | Seek guidance from experts in implementation and sustainability |  |
|  |  |  | Visit other sites | Visit outlets or settings where healthy food retail practices have been successfully implemented or sustained, to observe, learn, and adapt strategies. |  |
|  |  |  | Other (inductively coded; semantic) |  |  |
|  | Train and educate stakeholders | | Conduct educational meetings | Hold meetings with relevant stakeholders (e.g., managers, supervisors, staff, volunteers, or community partners) to build knowledge and skills for implementing and sustaining healthy food retail practices. |  |
|  |  |  | Conduct educational outreach visits | Have a trained person meet with outlet staff on-site to provide tailored education and support on implementing and sustaining healthy food retail practices. |  |
|  |  |  | Shadow other experts | Provide opportunities for outlet staff or managers to observe and learn directly from others who have successfully implemented and sustained healthy food retail practices. |  |
|  |  |  | Conduct ongoing training | Plan and deliver regular training to support healthy food retail implementation and sustainment, including onboarding for new staff and refresher sessions for existing staff. |  |
|  |  |  | Create a learning collaborative | Facilitate groups of outlets or organisations coming together to share experiences, solve problems collectively, and foster a supportive environment for sustainment of healthy food retail practices. |  |
|  |  |  | Distribute educational materials | Distribute educational materials (including guidelines, manuals, and toolkits) in person or electronically to support implementation and sustainment of healthy food retail practices. |  |
|  |  |  | Review and update educational materials | Regularly review and revise resources (e.g., guidelines, menus, training materials) to ensure they remain up to date, practical, and relevant for sustaining healthy food retail practices. |  |
|  |  |  | Make training dynamic | Use varied and interactive training approaches tailored to different learning styles and outlet contexts to keep training engaging and practical. |  |
|  |  |  | Provide ongoing consultation | Provide ongoing consultation with one or more experts in the strategies used to support implementing and sustaining healthy food retail practices. |  |
|  |  |  | Use train-the-trainer strategies | Train designated staff or partner organisations who can then train others within outlets to implement and sustain healthy food retail practices. |  |
|  |  |  | Work with educational institutions | Encourage educational institutions to deliver (healthy food retail) education and build workforce capacity for implementing and sustaining healthy food retail practices. |  |
|  |  |  | Other (inductively coded; semantic) |  |  |
|  | Support retailers | | Develop resource sharing  Agreements | Develop partnerships with organisations that can share resources, tools, or infrastructure to support outlets in implementing and sustaining healthy food retail practices. |  |
|  |  |  | Facilitate relay of data to retailers | Provide timely and accessible data (e.g., sales figures, customer feedback, compliance reports) to retailers using simple communication channels that encourage use for decision-making. |  |
|  |  |  | Remind retailers | Develop or use systems (e.g., prompts, reminders, checklists) to help retailers and staff remember and act on healthy food retail guidelines or commitments. |  |
|  |  |  | Revise professional roles | Adapt and expand staff or volunteer roles to include responsibilities for promoting and sustaining healthy food retail practices. |  |
|  |  |  | Other (inductively coded; semantic) |  |  |
|  | Engage consumers | | Increase demand | Use strategies (e.g., promotions, incentives, menu highlights) to boost consumer interest and demand for healthier food and drink options. |  |
|  |  |  | Intervene with consumers  To enhance uptake and adherence | Develop strategies with consumers to encourage and problem solve around adherence to healthy food retail practices. |  |
|  |  |  | Involve consumers and family members | Include consumers, families, or community members in efforts to design, implement, and sustain healthy food retail practices. |  |
|  |  |  | Prepare consumers to be active participants | Prepare consumers to be active participants by  Equipping them with knowledge, skills, and confidence to ask about healthy options and make informed choices. |  |
|  |  |  | Use mass media | Use media campaigns (e.g., social media, local newspapers, radio) to reach large audiences and promote healthy food and drinks. |  |
|  |  |  | Other (inductively coded; semantic) |  |  |
|  | Utilise financial strategies | | Access new funding | Access new funding to facilitate the sustainment |  |
|  |  |  | Alter incentive / allowance  Structures | Use incentives or allowances (e.g., discounts, supplier deals) to encourage sustainment of healthier food retail practices. |  |
|  |  |  | Alter consumer fees (i.e. Use pricing strategies) | Adjust pricing so healthier options are more affordable, and less healthy items are relatively more expensive. |  |
|  |  |  | Develop disincentives | Introduce financial or operational disincentives for not implementing or sustaining healthy food retail practices (e.g., reduced eligibility for grants or contract renewals). |  |
|  |  |  | Fund and contract for healthy food retail practices | Use contracts, funding processes, or agreements that motivate and support retailers to deliver and sustain healthy food retail practices. |  |
|  |  |  | Other (inductively coded; semantic) |  |  |
|  | Change infrastructure | | Change accreditation or  Membership requirements | Adapt accreditation standards or membership requirements so that healthy food retail practices are required or encouraged. |  |
|  |  |  | Change compliance requirements  (change liability laws) | Strengthen compliance or accountability measures (e.g., government policies) that make retailers more willing or required to implement and sustain healthy food retail practices. E.g., moving from voluntary guidelines to mandatory policies. |  |
|  |  |  | Change physical structure and equipment | Evaluate periodically current configurations and adapt, as needed, the physical structure and/or equipment (e.g., refrigeration, shelving, display cabinets) to healthy food retail practices. |  |
|  |  |  | Change monitoring systems  (change record systems) | Revise monitoring or reporting systems to better track the implementation and sustainment of healthy food retail practices (if old systems insufficient) |  |
|  |  |  | Change food outlets or service locations | Change or expand food retail sites (e.g., moving kiosks, redesigning outlet spaces) to increase access to healthy options. |  |
|  |  |  | Create or change recognition/endorsement standards | Create or revise recognition or endorsement standards (e.g., certification schemes, quality marks) that formally acknowledge outlets meeting healthy food retail practices. |  |
|  |  |  | Mandate change | Have leadership bodies (e.g., councils, health departments, sporting associations, healthcare organisations, schools) declare healthy food retail practices a priority and set requirements for implementation and sustainment. |  |
|  |  |  | Start a dissemination organisation | Identify or establish an organisation responsible for disseminating and supporting ongoing delivery of healthy food retail practices (could be for-profit or not-for-profit). |  |
|  |  |  | Other (inductively coded; semantic) |  |  |

**References**

Backman, B., Blake, M. R., Zorbas, C., Chung, A., Schulz, S., Brown, V., Mäki, P., & Yoong, S. (2026). Sustaining healthy food retail: do Australian and New Zealand government policies consider long-term change? *Social Science & Medicine*, *390*, 118876. <https://doi.org/https://doi.org/10.1016/j.socscimed.2025.118876>

Goi, C. L. (2009). A Review of Marketing Mix: 4Ps or More? *International Journal of Marketing Studies*, *1*, 2-2. <https://doi.org/10.5539/ijms.v1n1p2>

Nathan, N., Powell, B. J., Shelton, R. C., Laur, C. V., Wolfenden, L., Hailemariam, M., Yoong, S. L., Sutherland, R., Kingsland, M., Waltz, T. J., & Hall, A. (2022). Do the Expert Recommendations for Implementing Change (ERIC) strategies adequately address sustainment? [Original Research]. *Frontiers in Health Services*, *2*. <https://doi.org/10.3389/frhs.2022.905909>

Shelton, R. C., Cooper, B. R., & Stirman, S. W. (2018). The Sustainability of Evidence-Based Interventions and Practices in Public Health and Health Care. *Annual Review of Public Health*, *39*(1), 55-76. <https://doi.org/10.1146/annurev-publhealth-040617-014731>

1. The Integrated Sustainability Framework by Shelton et al. (2018) integrates theoretical and empirical insights on factors influencing the sustainability of health interventions, categorised into outer context (e.g., sociopolitical), inner context (e.g., organisational resources), intervention characteristics (e.g., perceived benefit), interventionist factors (e.g., skills), and processes (e.g., training). This framework has previously been adapted for healthy food retail policy analysis (Backman et al., 2026). [↑](#footnote-ref-1)
2. Sustainment-explicit Expert Recommendations for Implementing Change by Nathan et al. (2022) offers a comprehensive glossary of strategies to support adoption, implementation and sustainment, categorised by these three different phases. [↑](#footnote-ref-2)
